# Oxaliplatin and Bolus-Modulated 5-Fluorouracil as a Second-Line Treatment for Advanced Pancreatic Cancer: Can Bolus Regimens Replace FOLFOX When Considered for Second Line?

**DOI:** 10.1155/2013/358538

**Published:** 2013-02-24

**Authors:** A. Azmy, S. Abdelwahab, M. Yassen

**Affiliations:** ^1^GIT and Gynecological Oncology Unit, Ain Shams University, Cairo 11539, Egypt; ^2^Department of Clinical Oncology, Ain Shams University, Cairo 11539, Egypt

## Abstract

*Objective*. Comparing activity of 2 regimens combining oxaliplatin to bolus modulated fluorouracil as second line treatment in advanced pancreatic adenocarcinoma pretreated with gemcitabine-containing schedule. *Methods*. Forty eight patients with advanced pancreatic adenocarcinoma were randomly assigned to receive either FU 500 mg/m^2^ IV bolus weekly ×6 weeks plus leucovorin 500 mg/m^2^ IV weekly for 6 weeks during each 8-week cycle plus oxaliplatin 85 mg/m^2^ IV on weeks 1, 3, and 5 of each 8-week (FLOX) OR receive weekly intravenous infusions of oxaliplatin 40 mg/m^2^, 5-FU 500 mg/m^2^, and leucovorin 250 mg/m^2^ (3 weeks on, 1 week off). *Results*. Non progression(PR+SD) was found in 33.5% for first regimen and 29% for second regimen, and 37.5% had *clinical benefit* (FLOX regimen) compared to 50% in 3-weeks regimen. The median TTP was 3.9,4 months respectively. Median OS was 8, 9 months for both regimens. Only one case in 3-weeks arm suffered from grade IV diarrhea. Two cases > grade 2 neutropenia were observed; one in each treatment groups. Grade 3 anemia was recorded in 3 patients (2 in FLOX arm, one in 3-weeks arm). *Conclusions*. Both regimens showed encouraging efficacy, acceptable toxicity, and clinical benefit.

## 1. Introduction

Due to the fact that the majority of pancreatic cancers are unresectable upon diagnosis, curative intent is rarely a goal of treatment, rather increasing survival, time to progression, and quality of life are more realistic goals. Without treatment, median survival for patients with an advanced stage of disease ranges from 3 to 4 months, whereas in patients receiving chemotherapy with single-agent gemcitabine, median survival times between 4.9 and 7.2 months have been reported in randomized phase III studies [1]. Gemcitabine has been the solo player in the field of pancreatic cancer, treatment after replacing 5-FU since 1997, and is still regarded as one standard of care for the first-line systemic chemotherapeutic treatment of patients with advanced pancreatic cancer worldwide. So far, only two randomized phase III trials have demonstrated a significant prolongation of survival with the use of gemcitabine-based combination therapy with either erlotinib or capecitabine [2]. Eventually, progression will occur and the real challenge will be how to treat a patient with advanced pancreatic cancer failing to respond or progressing after gemcitabine. There is no evidence-based treatment recommendation for these patients. The National Comprehensive Cancer Network guidelines for pancreatic adenocarcinoma currently recommend second-line chemotherapeutic treatment after gemcitabine failure in selected patients using, for example, single-agent capecitabine or a combination therapy of fluorouracil, leucovorin and oxaliplatin (FOLFOX-) like regimen [3]. To date, there is no large randomized trial confirming the survival advantages of second-line chemotherapy over best supportive care, yet the preliminary results from a small randomized German study comparing BSC alone versus 5-FU, folinic acid, and oxaliplatin plus BSC after gemcitabine failure showed a prolongation of median survival by approximately 2.6 months with the use of chemotherapy (2.3 versus 4.9 months) [4]. These data were supported by a Japanese study that reported a median survival time of approximately 1.9 months after failure of first-line gemcitabine in 74 patients with pancreatic cancer (of whom 97% received no second-line treatment) [5]. Many protocols containing oxaliplatin, 5FU, and leucovorin, as FOLFOX, FLOX, and 3-week bolus 5FU plus leucovorin and oxaliplatin, are known. Preclinical data suggested that the 5-FU plus oxaliplatin combination is more cytotoxic when 5-FU is given as a short exposure [6], which gives a rationale for exploring the toxicity and efficacy of such protocols in advanced pancreatic cancer. In the current study, we conducted a randomized trial to compare two protocols; FLOX and the 3-weeek bolus protocol regarding toxicity, response rate, and time to progression as primary end points, then overall survival as secondary endpoints.

## 2. Patients and Methods

Patients with advanced unresectable or metastatic pancreatic adenocarcinoma were enrolled under the following Eligibility criteria.

*Inclusion Criteria*. (I) Patients with histologically or cytologically proven locally advanced or metastatic pancreatic adenocarcinoma, (II) with at least 1 bidimensionally measurable lesion (World Health Organization (WHO) criteria); (III) Eastern Cooperative Oncology Group (ECOG) PS of 1-2; (IV) tumor progression after first line gemcitabine (whether gemcitabine pretreated or gemcitabine resistance); (V) absence of severe uncontrolled cardiovascular, metabolic, infectious, or neurological diseases; (VI) adequate bone marrow reserve (neutrophil count > 1.5 × 10^9^/L, platelet count > 100.000/mm^3^ and Hb > 10 g/dL); (VII) adequate liver function (serum bilirubin < 1.5 mg/dL, serum transaminases < 2x the upper limit of normal); (VIII) adequate renal function (serum creatinine < 1.5 mg/dL); (IX) and age between 18 and 75 years. All participating patients were required to give written informed consent, and ethical approval from MOC committee was obtained before the start of the whole procedure.

*Exclusion Criteria*

- Histologic types other than adenocarcinoma.
- Neuropathy ≥ CTCAE grade 1.
- Ototoxicity > CTCAE grade 2.
- Serious, active comorbidity, including any of the following: unstable angina and/or NYHA class II–IV congestive heart failure requiring hospitalization within the past 12 months, transmural myocardial infarction within the past 12 months, acute bacterial or fungal infection requiring IV antibiotics, chronic obstructive pulmonary disease exacerbation or other respiratory illness requiring hospitalization or precluding study therapy, hepatic insufficiency resulting in clinical jaundice and/or coagulation defects, active gastrointestinal (GI) ulcers, GI bleeding, inflammatory bowel disease, or GI obstruction, Inadequately controlled hypertension, defined as systolic BP > 150 mm Hg and/or diastolic BP > 90 mm Hg on antihypertensive medications, serious cardiac arrhythmia on medication (well-controlled atrial fibrillation on medication allowed), and history of hypertensive crisis or hypertensive encephalopathy.

### 2.1. Pretreatment Evaluation

All patients were subjected to staging procedures consisted of medical history, physical examination, echocardiography, serum chemistry panel, complete blood picture, CEA, and CA 19-9. Extent of disease was determined by chest X-rays, computed tomography and/or nuclear magnetic resonance, and endoscopy as needed. Patients underwent followup examinations until death.

### 2.2. Randomization Procedures

Patients were randomly assigned to one of the treatment regimens (block randomization at 4), where 24 patients were enrolled for each treatment group.

### 2.3. Treatment

#### 2.3.1. FLOX Regimen

Oxaliplatin 85 mg/m^2^ was administered as a 2-hour infusion before LV and FU on days 1, 15, and 29 of the treatment cycle. LV 500 mg/m^2^ was administered as a 2-hour intravenous infusion weekly for 6 consecutive weeks (on days 1, 8, 15, 22, 29, and 36 of the treatment cycle), followed by a 2-week rest period. FU 500 mg/m^2^ was administered as an intravenous bolus 1 hour after the LV infusion was begun and was administered weekly for 6 weeks (on days 1, 8, 15, 22, 29, and 36 of the treatment cycle), followed by a 2-week rest period.

#### 2.3.2. 3-Week Bolus Regimen

2-hour intravenous infusion of oxaliplatin 40 mg/m^2^ was followed by bolus leucovorin 250 mg/m^2^ and bolus 5-FU 500 mg/m^2^. Each course consisted of weekly administrations for 3 consecutive weeks followed by a week of rest. Therapy continued until disease progression, unacceptable toxicity, patient's refusal, or a maximum of 6 courses.

All patients received intravenous dexamethasone 8 mg, Ondansitrone 8 mg as antiemetic prophylaxis. Therapy was withheld in case of a platelet count of less than 100.000/mm^3^ or a neutrophil count of less than 1.500/mm^3^ or for bilirubin greater than 1.5 times the upper reference level (URL) or transaminases greater than 3 times the URL. During the entire study period, patients received full supportive care to control pain or other symptoms, with careful recording of the treatment.

### 2.4. Toxicity

Adverse events were graded according to the National Cancer Institute common toxicity. Oxaliplatin was reduced in the event of persistent paresthesia/dysesthesia between cycles or with pain lasting for >7 days according to staff physician's decision. When paresthesia/dysesthesia with either pain or functional impairment persisted between cycles, Oxaliplatin was discontinued.

### 2.5. Evaluation and Statistical Methods

Measurable disease response was assessed by RECIST criteria [7]. Partial response (PR), stable disease (SD), and progressive disease (PD) were determined according to these criteria. The sum of PR and SD was reported as disease control rate (DCR). OS was estimated from the date of first treatment to the date of death or the last followup. Clinical benefit assessment was based on patients and physician-reported improvement of cancer-related symptoms and/or stabilization of improvement of PS. The TTP was calculated from the first treatment infusion to the first objective evidence of disease progression assessed by CT scan measurements or early death or date of clinical deterioration and patient not assessable for response. All patients with at least 1 chemotherapy administration were assessed for toxicity. Efficacy assessments were performed on patients who received at least 1 course of therapy. TTP and OS since the start of treatment were estimated on an intent-to-treat basis and analyzed according to the Kaplan-Meier method. Comparison between survival curves was done through log rank test to estimate *P* value utilizing GraphPad prism version 5 software. The required number of patients for this phase II study was determined according to a Jehan phase II optimal design [8] for a goal of 20% true clinical benefit; with *α*- and *β*-error probability of 0.05 and 0.20, respectively, an accrual of 24 patients assessable for response was planned.

## 3. Results

Forty-eight patients with unresectable or metastatic pancreatic cancer pretreated with gemcitabine (including gemcitabine resistance or gemcitabine pretreated) in Ain Shams University Hospitals were included along the period between October 2008 and September 2011.

The patients' characteristics encountered in the current study were outlined in Table 1.

Median age for both groups was 56 years and 54 years, respectively. Sixteen males out of total twenty-four cases were encountered in FLOX arm compared to 17 in 3-week bolus arm. Thirty patients in both groups received prior Gemcitabine as a single agent.

### 3.1. Toxicities

Grade 3 or 4 toxicities experienced by at least 5% of patients according to treatment arm are summarized in Table 2.

Only one case in 3-week arm suffered from grade IV diarrhea. Two cases of neutropenia exceeding grade 2 (but no febrile neutropenia) were observed; one in each treatment groups. Grade 3 anemia was recorded in 3 patients (2 in FLOX arm, one in 3-week arm). Most nonhematological side effects were less than grade 3.

### 3.2. Efficacy

No complete response was registered among all assessable 48 patients throughout the study duration *for FLOX regimen*, three patients (12.5%) had partial response, five patients (21%) had stable disease, and three out of 8 patients with pain at presentation (37.5%) had clinical benefit. The median time to progression was 3.9 months (95% CI, 2–4.6) (range: 1.5–5.5). Median survival time was 8 months (95% CI, 4.0–12).

**Figure 1:**
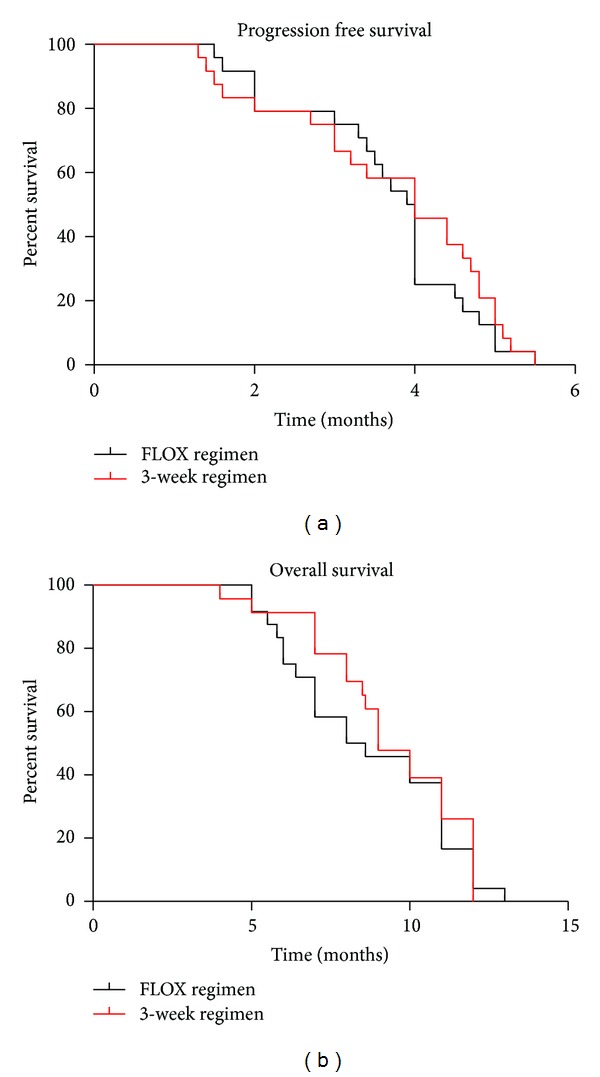
(a) Progression-free survival; Kaplan-meier curves of both treatment regimens. (b) Overall survival; Kaplan-Meier curves of both treatment regimens.

*For 3-week regimen*, two patients (8%) had partial response, five patients (21%) had stable disease, and four out of 8 patients with pain at presentation (50%) had clinical benefit as shown in Table 3. The median time to progression was 4 months (95% CI, 1.8–5) (range: 1.2–6). Median survival time was 9 months (95% CI, 3.5–13) as shown in Figures 1(a) and 1(b).

There was no statistical significance in progression-free survival between the 2 regimens (*P* value by log rank test = .4619), and so was the situation in overall survival (*P*-value by log rank test = .5248).

### 3.3. Cost Comparison

Although it was not planned as a target for the current study, yet it was an interesting issue to compare cost of chemotherapy per patient for every 8 weeks of treatment for each regimen. For FLOX regimen this cost was approximately 1200 USD versus 1400 USD for the 3-weeks regimen (due to mainly the amount of discarded oxaliplatin in every injection time that was more in the second regimen) as in Table 4.

## 4. Discussion

Advanced pancreatic cancer remains a rapidly lethal cancer, with a median survival of 6 months with currently approved therapies [8]. The role of second-line chemotherapy after failure of first-line therapy in such cases is not well established, but a theoretical possibility exists in which salvage chemotherapy after the failure of first-line treatment may influence the survival. For the scale of patients with good performance status, progressing after first-line gemcitabine therapy, NCCN recommends fluoropyrimidine-based chemotherapy [9]. But for time being there is still a debate to treat or best supportive care? A phase III trial after failure of first-line gemcitabine compared BSC plus with biweekly oxaliplatin combined with weekly 5-FU as 24 hours infusion plus leucovorin, versus BSC alone [4]. After the first 46 patients out of 165 planned, the BSC arm had to be closed because BSC alone was no longer accepted by participating centers, with a possible survival benefit for second-line chemotherapy: 21 weeks (95% CI; .7; 23.3) versus 10 weeks (95% CI; 7.7; 12.3). So a second question is as the following: *what is the best option of treatment?* Adding oxaliplatin to continuous infusion fluoropyrimidine as a second-line salvage therapy for this category has been investigated in some phase II trials [10–12]. In a series of unselected patients the FOLFOX4 regimen yielded a 14% PR rate with 38% of patients showing SD for a DCR of 57%. Median duration of PR was 5.2 months, while median time to progression and overall survival was 4 and 6.7 months, respectively [10], but all the regimens of continuous infusion necessitate either hospitalization or pump application with their financial load upon health care system. This was the rationale to investigate regimens including oxaliplatin and bolus fluorouracil, with the theoretical premise of being as active as continuous infusion regimens, as well simpler in administration, less in cost, and better in toxicity profile. In the current study, two regimens of oxaliplatin and bolus fluorouracil have been investigated, FLOX regimen that was used in metastatic colorectal cancer with adequate efficacy and acceptable toxicity profile [12]. The current study revealed *nonprogression* (PR + SD) in 33.5% for first regimen and 29% for second regimen, and 37.5% had *clinical benefit* (FLOX regimen) compared to 50% in 3-week regimen. The median time to progression was 3.9 months and 4 months, respectively. Median survival time was 8 months and 9 months for both regimens, respectively, with no statistically significant difference in progression-free or overall survival. Regarding toxicity, only one case in 3-week arm suffered from grade IV diarrhea. Two cases of neutropenia exceeding grade 2 (but no febrile neutropenia) were observed; one in each treatment group. Grade 3 anemia was recorded in 3 patients (2 in FLOX arm, one in 3-week arm). Most nonhematological side effects were less than grade 3.

So it is the time for the 3rd question; are these results comparable to those of FOLFOX regimens (infusion fluorouracil)? Gebbia et al., 2007, carried out a retrospective study including 42 patients who received standard FOLFOX4 regimen biweekly until progression or unacceptable toxicity. The study revealed six partial responses (14%) and 16 stabilizations (38%) were recorded for a tumor growth control rate of 57%. The median time to progression (TtP) was 4 months (range 1–7 months), and median overall survival (OS) was 6.7 months (range 2–9 months). A stabilization of performance status (PS) and a subjective improvement of cancer-related symptoms was recorded in 27 patients [13]. The good nonprogression rate in this study may be attributed to the high percentage of responding patients in this study to the first line therapy (50%) compared to 12% in our study.

Tsavaris et al., 2005, in a prospective Phase II study evaluated a second-line combination regimen of oxaliplatin together with leucovorin-modulated 5-FU in 30 patients and revealed an encouraging response rate of 23% with a corresponding disease-control rate of 53%. Median overall survival was 5.8 months in this patient population [14]. These data correlate well with the retrospective analysis from Italy, which found a response rate of 14% together with a disease-control rate of 52% with the use of a FOLFOX-4 regimen in gemcitabine-pretreated patients [14].

Another phase II trial of oxaliplatin plus capecitabine in a series of 41 patients reported a PR in one case and SD in eight patients with a median OS of 5.8 months, and a 6-month and 1-year survival rate of 48% and 22%, respectively [9]. Toxicity was, however, significant. Preliminary results of another trial of OXP/5-FU in a series of 23 patients have shown an OS of 4 months [15].

Novarino et al., 2009, in a study on 23 gemcitabine pretreated patients with advanced pancreatic cancer revealed no objective response in all 17 assessable patients and 4 patients had stable disease, whereas 13 had tumor progression. Median duration of stable disease was 14 weeks. Median time to progression of disease (TTP) was 11.6 weeks. Seven patients experienced grade 3-to-4 toxicity. The regimen was associated with 36% clinical benefit [16].

In conclusion, combining oxaliplatin to bolus fluorouracil (either in FLOX or 3-week regimens) as a second line in gemcitabine pretreated patients with advanced or metastatic pancreatic adenocarcinoma showed encouraging efficacy, acceptable toxicity, and some clinical benefit specially when palliation or good quality of life is a target keeping in mind the simplicity in administration, the no need for hospitalization, and the less financial load specially with FLOX. Further studies with large number of patients investigating the efficacy and tolerability of such bolus regimens in gemcitabine-pretreated pancreatic cancer patients are warranted.

## Figures and Tables

**Table 1:** Patients' characteristics.

Characteristics	FLOX arm	3-week arm	*P* value
Age			
Median	56	54	.45
Range	44–69	41–68	
Gender			
Male	16 (67%)	17 (71%)	.49
Female	8 (33%)	7 (29%)	.35
Disease at presentation			
Locally advanced	15 (63%)	14 (58%)	.44
Metastatic	9 (37%)	10 (42%)	.23
Site of metastases			
Liver	5 (21%)	6 (25%)	.48
Lung	1 (4%)	1 (4%)	—
LN	2 (8%)	2 (8%)	—
Peritoneal	1 (4%)	1 (4%)	—
others	—		
Previous surgery			
Palliative	5 (21%)	4 (17%)	.3
Radical	1 (4%)	1 (4%)	—
None			
Prior chemotherapy			
Gemcitabine single agent	14 (58%)	16 (67%)	.23
Gem + 5FU	8 (34%)	6 (25%)	.17
Gem + Platinol	2 (8%)	2 (8%)	—
Response to prior therapy			
Partial response	—	—	
Stable disease	2 (8%)	3 (12.5%)	.12
Progression	22 (92%)	21 (87.5%)	.27
Presented symptoms			
Pain	8 (34%)	8 (34%)	—
Weight loss	20 (84%)	20 (84%)	—

**Table 2:** Patients with grade 3 or 4 toxicity by the NCI Common Toxicity Criteria version 3.0.

Characteristics	FLOX arm		3-week arm		*P* value
	Grade III	Grade IV	Grade III	Grade IV
Diarrhea	4 (16%)	1 (4%)	4 (16%)	1 (4%)	.11
Dehydration	2 (8%)	—	1 (4%)	—	.09
Nausea	1 (4%)	—	2 (8%)	—	.101
Vomiting	3 (12%)	—	1 (4%)	—	.07
Stomatitis	1 (4%)	—	1 (4%)	—	—
Hematological					
Neutropenia	1 (4%)		1 (4%)	—	—
Anaemia	2 (8%)		1 (4%)	—	.089
Thrombocytopenia	—	—	—		
Neurosensory	1 (4%)	—	1 (4%)	—	—
Thrombosis and embolism	1 (4%)	—	1 (4%)	—	—
Liver					
Elevated transaminases	2 (8%)	—	3 (4%)	—	.21

**Table 3:** Summarizing efficacy results of the 2 regimens.

	FLOX arm	3-week arm	*P* value
Partial responses	3 (12.5%)	2 (16%)	.23
Stable disease	5 (21%)	5 (21%)	.5
Clinical benefit	3/8 (37.5%)	4/8 (50%)	.19

**Table 4:** Cost comparison of the 2 regimens.

	FLOX arm	3-week arm	*P* value
Cost/patient/8 weeks	1200 USD	1400 USD	.013
